# The health Care Management of farmers who use pesticides in Northeast Brazil: use of pesticides and the Care Management

**DOI:** 10.1186/s12913-023-09782-0

**Published:** 2023-07-21

**Authors:** Astrid Merino Silverio, Maristela Casé Costa Cunha, Márlon Vinícius Gama Almeida

**Affiliations:** 1 Graduate Program in Human Ecology and Socioenvironmental Management, State University of Bahia CAMPUS III: DTCS - Campus III, Av. Edgard Chastinet Guimarães, s/n, São Geraldo, Juazeiro, Bahia 48.904-711 Brazil; 2 Department of Biological Sciences, University of Bahia CAMPUS VIII, R. da Gangorra, 503, Gen. Dutra, Paulo Afonso, Bahia 48608-240 Brazil; 3 Department of Family and Community Medicine, University of Vale do São Francisco (Univasf), Rua Aurora, s/n, Gen. Dutra, Paulo Afonso, Bahia 48605-560 Brazil

**Keywords:** Care networks, Integrality in health, Multiprofessional team, Pesticide damage, Public health

## Abstract

In Brazil, the health of communities that use pesticides is still neglected due to the lack of greater social understanding of damage to health and an insufficient care network. The objective of the research was to investigate health management practices, from the perspective of the expanded health care network (RAS), focusing on riverside farmers who use pesticides in the municipalities of Petrolina (PE) and Juazeiro (BA) in the Northeast of Brazil. The study aimed to explore these practices from the perspective of the healthcare network (RAS). This is a qualitative research, with a methodological framework based on the Grounded Theory in a constructivist way (CGT). Twenty-six health professionals and RAS managers participated, at the municipal and regional levels in Petrolina (PE) and Juazeiro (BA). Two sample groups were composed for data collection, carried out through semi-structured interviews. The analysis resulted in six categories, which, when integrated, made up the theoretical model “Model of care management for riverside farmers”. The RAS must have teams trained in the care of riverside farmers who use pesticides in primary care, and specialized teams in secondary and tertiary care, who are a reference. The theoretical model formulated considers that the RAS, strengthened in terms of diversity of actions and services, will provide better care management for farmers.

## Introduction

The care network and Care Management (CM) for riverside farmers who use pesticides are components of the National Health Network (RNS). In 2002, the RENAST (Integral Worker Health Care) was incorporated into primary and secondary healthcare networks as part of the strategy to organize actions in Workers’ Health across all levels of care. The integration of these services into the SUS (Unified Health System) network was made possible through the establishment of Specialized Reference Centers in Workers’ Health (CEREST). These centers aim to expand and integrate primary health care (PHC) services in communities [1, 2].

This network relates the types of care and the management of this care in the health of farmers in general and in agricultural communities1. The vulnerability of riverside communities is due to their complexity in relations with the environment and natural phenomena, as well as other public health hazards, such as river floods and communicable diseases [2]. Intoxications, deaths and injuries are frequent in farmers who use pesticides, but the absence of systematization in the recording of these procedures hinders the accuracy of data on their prevalence [3].

The notification of pesticide poisoning is compulsory, and must be performed in the face of suspicion or confirmation of disease or injury, and can be made by physicians, other health professionals or responsible for health facilities, public or private, according to Ordinance GM/MS No. 204, of February 17, 2016 [4].

Agricultural pesticides are defined by laws in Brazil (Federal Law No. 7,802 of July 11, 1989; State Law-Pernambuco (PE) No. 12,753, of January 21, 2005; State Law-Bahia (BA) No. 6,455, of January 25, 1993), such as: products whose purpose is to alter the composition of flora or fauna, to guard it from the harmful action of organisms considered harmful; as well as substances and products applied as defoliants, degerming agents, stimulants and growth inhibitors [5–8].

The health care of farmers in health services should consider the insertion of these in production processes, while recognizing work and modes of production, such as the use of pesticides in their crops, which define the health-disease-care process. Thus, it is necessary that the Family Health Teams (ESF) in the Basic Health Units (UBS) know the work or occupation of the user-worker and incorporate this knowledge to the actions of promotion, protection and surveillance, care and rehabilitation, in the care networks of the Unified Health System (SUS) [3].

Studies conducted in the state of Pernambuco [7] and Bahia [8] confirm that there is great exposure of rural workers to agricultural pesticides and highlight situations such as: ignorance of risk factors, non-use of Personal Protective Equipment (PPE), carcinogenic potential of the substances used, among others [9, 10].

The municipalities of Petrolina (PE) and Juazeiro (BA) have a very similar population of riverside farmers in the San Francisco river hydroterritory of 7,000 farmers. It is divided by communities that are assisted in health units, at the level of primary care and some points of supply at the level of secondary care (AS, e.g. Polyclinics) and CEREST. The people responsible for the care of the riverside farmer who uses pesticides are the Health Department (SSM), and the UBS under the management of the professional (coordinator of the UBS) selected by them [11, 12].

Even though two municipalities are so close and using the same pesticides, Juazeiro over the last few years has decreased the number of notifications for annual injuries when compared to the municipality of Petrolina [13]. Thus, how are the services being made in the health care networks of riverside farmers who use pesticides in the municipalities of Petrolina (PE) and Juazeiro (BA)?

If the impact on health by the use of pesticides is a problem not yet solved in Brazil [14], the health of communities that use pesticides is further neglected due to the lack of greater social compression of health damage and an insufficient care network. The objective of the research was to investigate health management practices, from the perspective of the expanded health care network (RAS), focusing on riverside farmers who use pesticides in the municipalities of Petrolina (PE) and Juazeiro (BA) in the Northeast of Brazil. The study aimed to explore these practices from the perspective of the healthcare network (RAS).

This study contributes to the progression of theoretical knowledge by visualizing the network of management and care, with the aim of developing an explanatory theoretical model based on empirical data and its limitations in the functioning of the integral service, for the planning of managers. New studies can mitigate health damage by the use of pesticides working in the field of human behavior in different spheres where the riverside farmer needs an adequate and fair social environment.

## Material and method

This is a cross-sectional study, with a qualitative, exploratory and descriptive approach to understand the actions and functioning of the health Care Management network of riverside farmers who use pesticides, in each of their service points. Having as methodological reference the Grounded Theory (CGT), opting for the use of the constructivist current of knowledge, proposed by Charmaz [15].

The data collection developed from January to May 2022, in the municipalities of Petrolina (PE) and Juazeiro (BA), northeastern Brazil, in the places of attention of the RNS (Interstate Health Care Network of the Middle Valley Of San Francisco, PE and BA, 2011), which carry out actions and are configured as primary and secondary care services, under the management of these municipalities and the States of Bahia and Pernambuco; Petrolina and Juazeiro function as reference microregions of worker health care for their states. The Health Units chosen as the gateway to the management network and the care of riverside farmers were: The Basic Health Unit (UBS) Josefa Bispo de Almeida in Petrolina and the Family Health Unit (USF) NH4/ Bonita – São José in Juazeiro.

In each municipality, the research team conducted interviews with various healthcare professionals. These included the doctor from the chosen Family Team, the head nurse, a nursing technician, a medical student in their final year, and two Community Health Agents. Additionally, interviews were conducted with healthcare professionals from CEREST, including the worker’s doctor, the worker’s nurse, the head coordinator of CEREST, a physiotherapist, and a psychologist. The research team also interviewed the doctor on duty at the Emergency Care Unit and a local dermatologist from the municipal consultation of Clinical Specialties.

The participants included in the study consisted of: 26 (13 in each group) health professionals and managers, workers in the municipal and state RNS of Petrolina and Juazeiro in the various points of care located in PHC and outpatient secondary care in each municipality, as well as in the municipal and state health departments, with at least one year of care experience. The signing of the free and informed consent form of the research was also requested and the willingness to answer the questions of the interview. However, professionals and managers who were on vacation or on leave were excluded from the research during the period of data collection of the points of the network visited.

The focus of the interview in this text is to gather information and insights about the healthcare practices and management related to riverside farmers who use pesticides. The questions aim to explore various aspects of care, including the interviewees’ experience, the specific actions taken for this care, the use of injury notification forms, the characteristics of care provided, the role of Primary Health Care/Basic Health Units (PHC/UBS) in managing care for these farmers, and the relationship between the care for riverside farmers who use pesticides and other components of the National Health Network (RNS).

At the end of the interview, the relationship points that the networks of the municipalities had to support the riverside farmers inside and outside the SUS were asked. This provides the visualization of the network, in addition to the functions of each point of assistance, to draw a representative figure of this process, which in the literature was not found.

The explanations of the functioning of the management network and the care of farmers were related to the role of the other points of attention, as well as the understanding of managerial aspects, in addition to care in a care network [16].

**Fig. 1:**
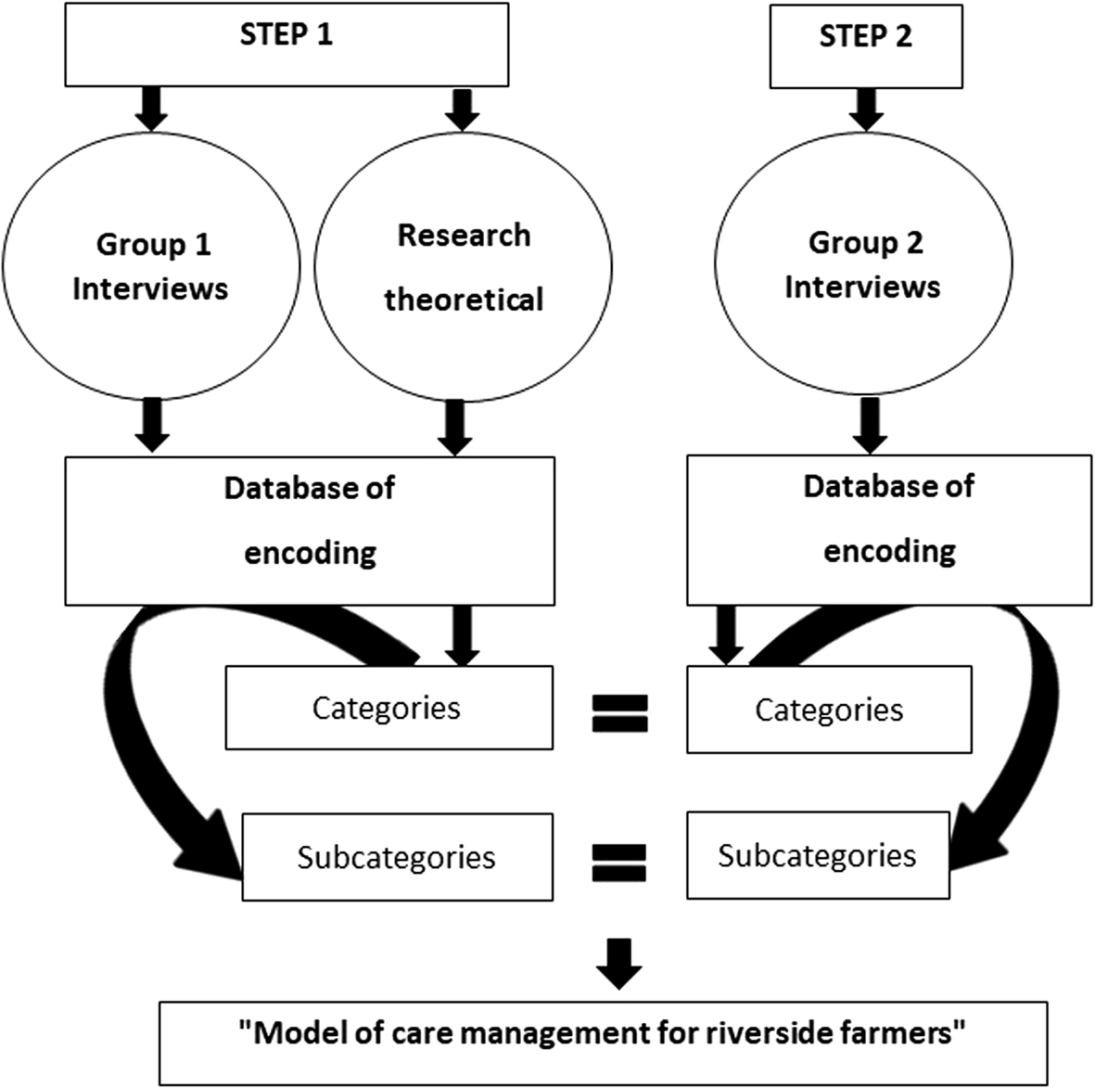
Flowchart of research Methodology Based on Constructivist Grounded Theory (CGT)

The data were coded in categories that represented the parts of the process and subcategories that explained them [15]. Thus, this approach was carried out the study of the care management network of riverside farmers, little researched in the scientific literature (See Fig. 1).

In the initial codification the choice was the group of interviews in the municipality of Petrolina. The excerpts of the interviews were thoroughly analyzed, and initial codes resulted from them, which were reformulated until the last interviewee. The categories emerged from the product of the constant comparative method, establishing analytical distinctions and making comparisons at each level of functioning in care management. The CGT comprises some coding steps to achieve the theoretical model [15].

The second stage consisted of focused coding, in which the codes are more directed, selective and conceptual when compared to the initial coding. During the focused coding process, the subcategories emerged, from the aggregation of the initial codes, by the continuous process of comparison of the analyzed data [15].

The third stage performed in data analysis was theoretical coding, in which the categories and subcategories that emerge from the codes are formed and integrated in order to expose the phenomenon or the central category. The validation of the theoretical model was performed with the participants of the second sample group, in order to discuss its resonance, each level of functioning and the veracity of the knowledge of each group [15] in the management network and the care of the riverside farmer [17], due to the health risk that pesticides cause [5, 9].

The research followed the guidelines and regulatory norms of the research involving human beings that make up Resolution 466/12, first obtaining the approval of the municipal bodies for its realization, with subsequent submission and approval of the research project in the Ethics Committee on Research with Human Beings of the State University of Bahia (UNEB) - opinion number 5.181.184 of December 21, 2021.

## Results

The collection and analysis of the data of the interview resulted in six categories, which comprised the theoretical model “Model of Care Management for riverside farmers” within the RNS, in RENAST and primary health care. This is considered the central category of the theoretical model, which is composed of the integration of 6 categories, from 26 subcategories (of the 26 participants) and 156 conceptual codes (the 156 answers). The Table 1 presents the analytical categories and subcategories, components of the care network model at different levels.

**Table 1:** Analytical categories and subcategories, components of the *Model of Care Management for riverside farmers* from the perspective of the health care network of the municipalities Petrolina- PE and Juazeiro-BA, 2022

**Categories**	**Subcategories**
*1. Knowing the functioning and points of the Care Management network (CM) of the riverside farmer who uses pesticides*	Recognizing CM as comprehensive health care for the riverside farmer who uses pesticides • Relating the *CM* to the planning of the care of the riverside farmer who uses pesticides • Unfolding the *CM* of the riverside farmer who uses pesticides in care and management dimensions • Understanding the CM as the responsibility of the health team, the riverside farmer who uses pesticides and his family • Basing the *CM* on the riverside farmer who uses pesticides in professional autonomy, knowledge and good care practices • Performing the *CM* to the riverside farmer who uses pesticides with the support of the management bodies and public health policies
*2. Knowing the riverside farmer who uses pesticides in the professional practice of the doctor of the Family Health Teams (ESF) since his graduation.*	Knowing the instruments for performing the care of the riverside farmer who uses pesticides • Identifying the home visit as ideal for the knowledge of the riverside farmer who uses pesticides • Recognizing the riverside farmer who uses pesticides as users of the services offered in health units • Identifying potentialities in the care of the riverside farmer who uses pesticides • Identifying weaknesses in the care of the riverside farmer who uses pesticides
*3. Knowing riverside that uses pesticides in the professional practice of community health agents (CHA)*	Knowing the instruments for performing the care of the riverside farmer who uses pesticides • Identifying the home visit as ideal for the knowledge of the riverside farmer who uses pesticides • Recognizing the riverside farmer who uses pesticides as users of the health services offered in the communities • Identifying potentialities in the care of the riverside farmer who uses pesticides • Identifying weaknesses in the care of the riverside farmer who uses pesticides
*4. Performing care to the riverside farmer who uses pesticides an integral part of the work process in Primary Care from the Basic Health Units (UBS) or Family Health Units (USF) and Secondary Health (AS) of specialist physicians*	Recognizing the importance of nursing consultation to perform care for the riverside farmer who uses pesticides in the Health Center and at home • Developing nursing care for the riverside farmer who uses pesticides in the nursing consultation at APS (Primary Health Care) • Developing care for the riverside farmer who uses pesticides in the emergence of Emergency Service Units (UPA) or Hospitals • Integrating Primary Health Care (PHC) with the health team (ESF) in caring for riverside farmers who use pesticides • Understanding the importance of permanent awareness in the theme of pesticide damage and the exchange of knowledge among professionals • Recognizing the link with the ESF as an important component for the care of the riverside farmer who uses pesticides
*5. Taking care of the riverside farmer who uses pesticides by the multidisciplinary team of Occupational Health Reference Center (CEREST)*	Defining the bond, continuity and coordination of care as a role of PHC • Understanding the integrality of care as collaboration and integration between health care points • Recognizing that the riverside farmer who uses pesticides plays his role in the care of • Establishing the role of CEREST in the care of the riverside farmer who uses pesticides
*6. Characterizing the role of health care points in care (RNS)*	• Defining the bond, continuity and coordination of care as a role of PHC • Understanding the integrality of care as collaboration and integration between health care points starting from the UBS •Knowing the riverside farmer who uses pesticides playing his role in the care •Establishing the role of CEREST in the care of the riverside farmer who uses pesticides

Source: elaborated by the authors

In this sense, for the CM to be consolidated, it is necessary the knowledge on the part of the professional, about how the farmer uses the pesticide, since the period of graduation and during his performance in health, performing the care at his point of attention, as well as in the referral of this person to the other levels of the RNS, according to the demands presented. For the regulation to happen properly, the points of attention must have their well-defined roles in the care of the riverside farmer who uses pesticides, in order to carry out the actions that compete with him.

These actions do not overlap, they happen in an integrated and circular way, restarting with the emergence of new situations, causing the CM to emerge to the riverside farmer who uses pesticides from the perspective of networked care, as a process in constant construction, coordinated by PHC.

In this research, the participants reinforced that the dimensions presented for the construction of the CM are at the same level of importance, as well as interconnect and share responsibilities, creating horizontal relationships between them. It is noteworthy that the CM should provide the planned care, through the use of available resources as are the CHA, and listing objectives, goals and strategies, manifesting itself as a creative and motivational process, with the intention of maintaining and favoring care, ensuring its quality to users and their families.

There are no mechanisms within the networks, during the study period, for performing molecular examination of the type of pesticide, which farmers would need in case they did not know the substance that causes the disease. Likewise, there is no multiprofessional team that performs specific rehabilitation care to the riverside farmer who uses pesticides, and the reference for cases of difficult management in primary care is limited. The number of vacancies of specialists, as in the case of dermatoses, the lack of dermatologists via SUS, make the regulation of these cases even more difficult. The medical specialists included in the research reported in the interview that: they are reserved in decisions about giving a clinical diagnosis that place pesticides as causing problems in human health by the political force that the ruralist bench has in government.

According to what the interviewees reported, the CM network in the SUS has the following flow relationships: the UBS as the entrance ports of the network, being the most important to achieve complete monitoring; the UPA and hospitals in cases of urgency or emergency; and even the same CEREST when the farmer is not well informed.

As weaknesses for the organization of care in RNS, the participants cited the numerous difficulties of communication and the disarticulation of information systems, which has generated informalpractices and the loss of important information for the care process [18].

**Fig. 2:**
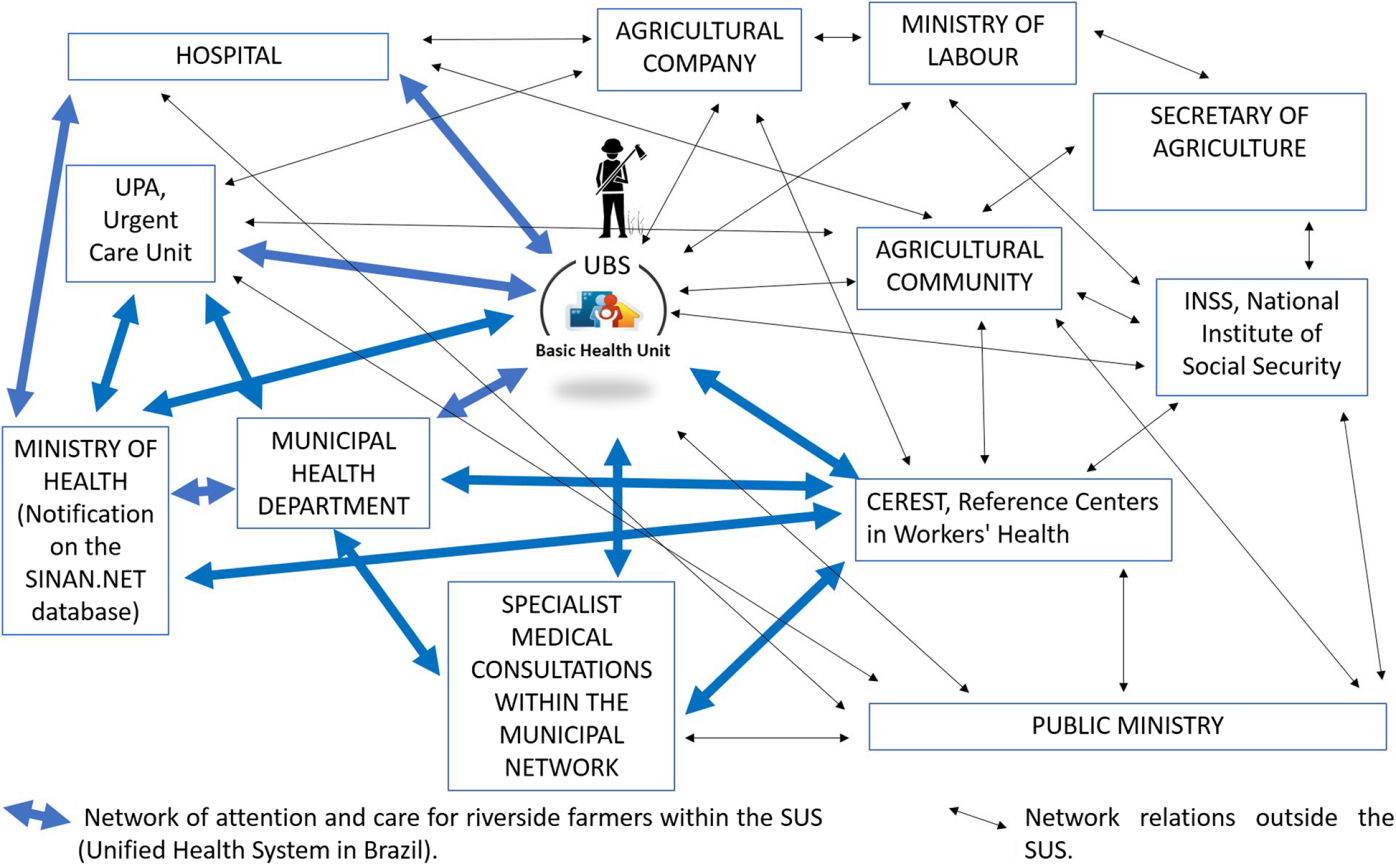
Municipal network model for Care Management (CM) of riverside farmers in pesticide care

In all these points of entry into the SUS network (Fig. 2) should happen the notification of the disease by the pesticide, with the completion of the form and sending to the health department from a dermatosis, dizziness, symptoms of depression, even severe intoxication or death. This fact was discussed in the interview given as insufficient the number of notifications at the entrance ports, and only through CEREST notification to obtain a real view of the problem. According to the reported by doctors, nurses and psychologists, participants of CEREST of each municipality it is the UBS that should always refer, study, diagnose and follow-up for the recovery of pesticide diseases.

Within the care network, there are relationships with other management centers from various spheres in society, which are also part of management and care. Among them: the Ministry of Labor, which assesses the need for a work safety staff and determines the quantity and periodicity of medical examinations and evaluations in large agricultural enterprises in the territory, although the small farmer does not have the relevant monitoring, both social as well as teaching, local and municipal supervision, to properly use the pesticide, although there are associations of farmers, entities in health, agriculture and the environment in the municipalities.

Also, the small farmer and those rural workers who have no official connection with agricultural companies have the ESF (with the CHA as protagonists) to monitor and trigger the health surveillance of health departments. This happens for the communities of the municipalities of Petrolina and Juazeiro; and probably because management policies follow the same guidelines, it is recurrent in many other Brazilian communities; because there is no proper functioning of the network due to a lack of capacity of the ESF.

## Discussion

In the model proposed by the research participants, the focus is on performing effective Care Management (CM) by strengthening the National Health Network (RNS) and implementing differentiated and qualified actions and services at each point. The aim is to improve services and meet the specific needs of riverside farmers who use pesticides, considering the complexity of their situations [19].

The process of notifying pesticide-related diseases in the Sinam Net involves collecting information from both public and private health centers through a notifiable form completed by the attending health worker. This document is transmitted and disseminated through a computerized network within the Epidemiological Surveillance System across all government levels (municipalities, states, and the federal government) to support investigation processes and provide data for analysis [3].

**Fig. 3:**
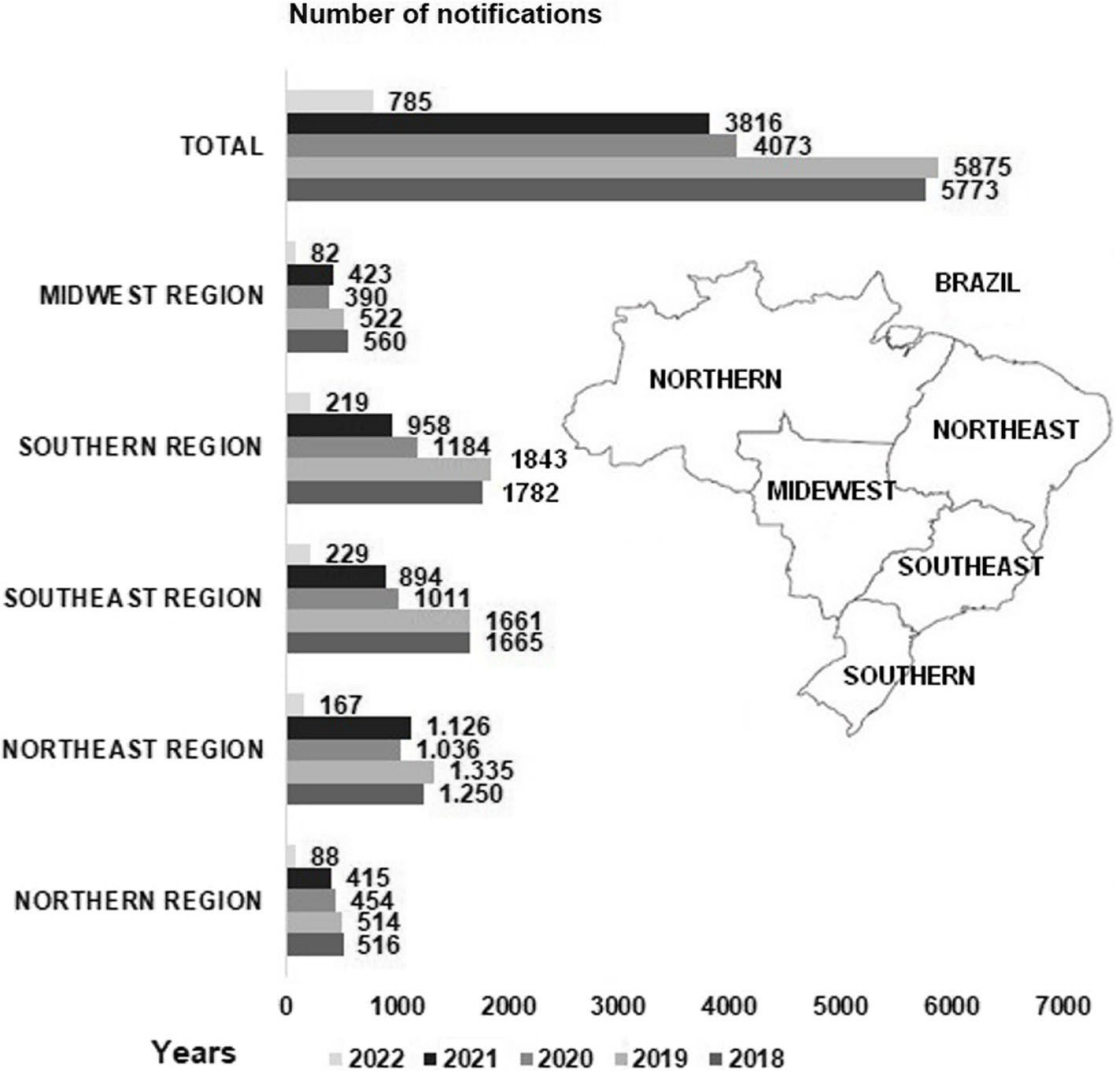
Injury notifications due to agricultural pesticide poisoning in Brazil’s regions from 2018–2022 (Sinan-Net)

**Fig. 4:**
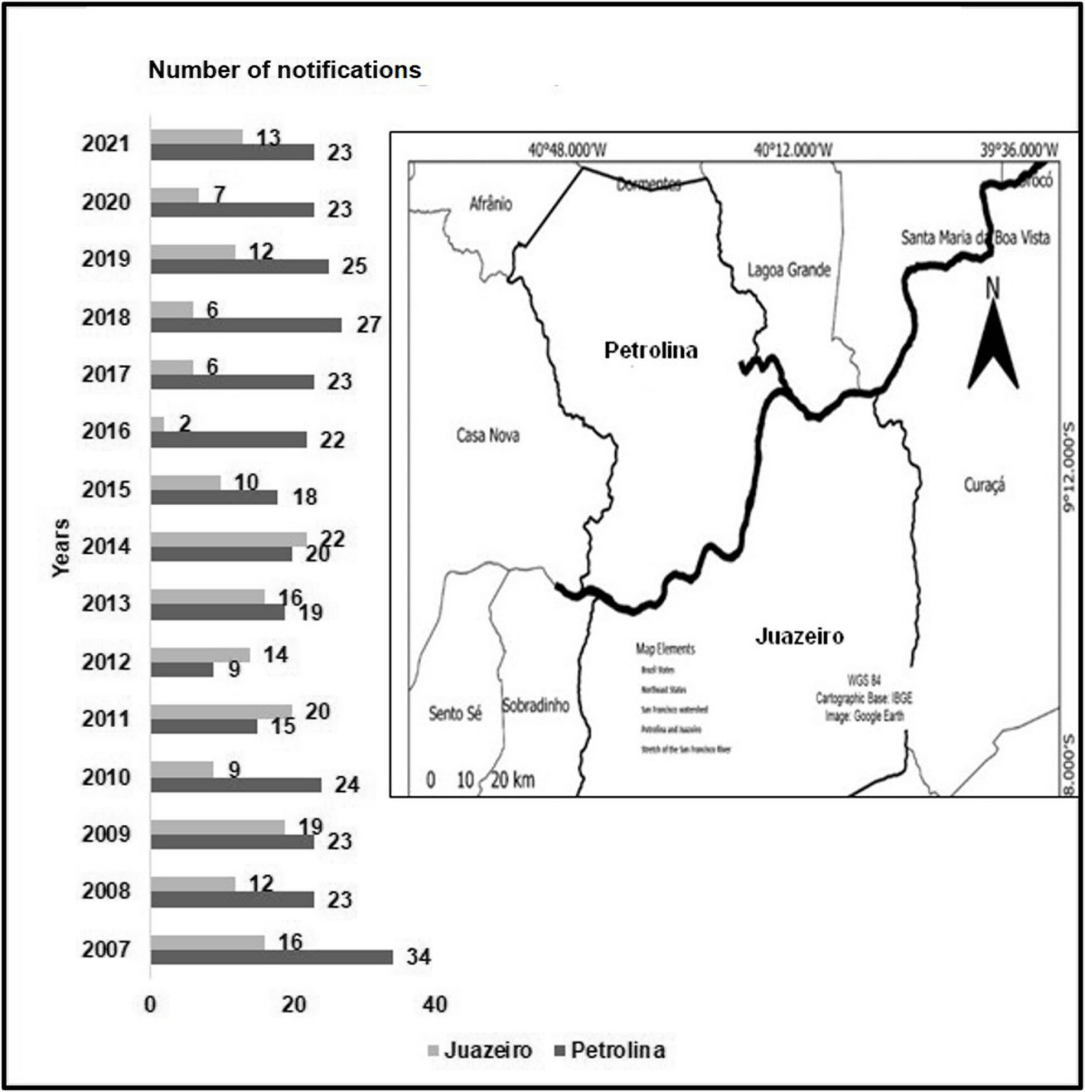
Injury notifications due to agricultural pesticide poisoning in Juazeiro/BA and Petrolina/PE from 2007–2021 (Sinan-Net)

Over the past five years, there has been a general decline in reporting cases of pesticide poisoning in most regions and federal entities in Brazil. However, it is worth noting that underreporting of cases and associated injuries has historically been significant [13] (Fig. 3). The Northeast region of Brazil has the highest number of agricultural pesticide-related cases, with the municipalities of Petrolina and Juazeiro showing the highest notifications within the San Francisco Valley region based on data from the Sinan Net system (Fig. 4).

There is a noticeable difference in the number of cases reported in the databases (Sinan-Net) between the municipalities of Petrolina and Juazeiro. Despite Juazeiro having a larger population of farmers, the number of reported cases is lower over the past 15 years (Fig. 4).

Comprehensive Public Health relies on CM by providing essential knowledge to healthcare personnel during their undergraduate education and professional practice. This enables efficient care delivery at various points of service and appropriate referrals of SUS (Unified Health System) users to other levels of the RNS based on their needs [19].

Resilience is also utilized to respond to new situations, allowing CM to continuously evolve for riverside farmers who use pesticides within the context of networked care, coordinated by Primary Health Care (PHC) [20, 21]. The CM should be understood as a human and social process that is based on interpersonal relationships, leadership, motivation, participation, communication and collaboration. Within the Scope of the RNS, the CM should be the product of the effective implementation of the mechanisms for coordinating care [3, 19–22].

The care provided to riverside farmers who use pesticides requires health professionals to reflect on the potentialities and weaknesses that individuals develop throughout the rehabilitation process. It is crucial to understand their ever-changing and diverse real needs, aiming for acceptance, harmonious coexistence, and a healthy outlook on their new situation [15, 23].

In the national management of networks like RENAST, the Ministry of Health plays a vital role in defining guidelines, regulating and coordinating actions, and providing political, financial, and technical support. This includes conducting studies and research based on priority criteria, strategically allocating resources, and addressing social demands. It is also essential to foster intersectoral collaboration with ministries such as Labor and Employment, Social Security, and the Environment to strengthen the comprehensive healthcare model for agricultural workers [1, 19].

The involvement of Family Health Teams at the Primary Health Care (PHC) level is considered strategic for the development of comprehensive care and has a positive impact on the quality of life of riverside farmers. Home visits and regular nursing consultations are important resources for their rehabilitation at all levels of complexity within the National Health Network (RNS), including PHC-based rehabilitation actions [3, 19, 20].

Collaborative care among Primary Health Care (PHC) team members, including the Family Health Support Center (NASF), emphasizes the importance of an interdisciplinary approach that values diverse perspectives in addressing the needs of riverside farmers who use pesticides. The central focus of PHC is to promote self-care and prevent complications for these individuals, empowering them and improving care for their families. Active participation from the Family Health Strategy (ESF) and the community is essential for enabling social reintegration, addressing health damages, enhancing risk perception, and improving overall quality of life [3, 19, 22, 23].

In secondary healthcare settings, the multidisciplinary team responsible for caring for riverside farmers who use pesticides in hospitals, Urgent Care Units (UPAs), and CEREST (Specialized Reference Centers in Workers’ Health) plays a crucial role. These teams are structured according to relevant ordinances [19], and their collaboration helps consolidate complementary structures of the Unified Health System (SUS) within municipal healthcare services for riverside farmers who use pesticides.

The CEREST team not only engages in rehabilitation activities but also serves as a knowledge hub, responsible for stimulating and developing actions related to continuing health education. They provide guidance to riverside farmers, their family members, and other healthcare professionals at different levels of care. This collaboration helps strengthen the relationship between CEREST and the ESF, ensuring the dissemination of important information and best practices [19].

The literature confirms that effective collaborations and connections between professionals and services are vital for ensuring continuity of care. The organization of care within the National Health Network (RNS) promotes universal, equitable, and comprehensive access, based on the principles of the SUS, through regionalization and hierarchization of services [1, 17, 19, 20]. However, challenges such as limited financial resources, decentralization of essential services, and difficulties with access and coordination can impede effective management and care [18].

Maintaining a well-functioning reference system, counter-referral system, and effective epidemiological surveillance through pesticide-related injury notifications are crucial. However, these processes are often hindered by poor completion of forms by healthcare professionals. The use of electronic medical records can be valuable in disseminating information among professionals, but in this research, it is primarily utilized by municipal-managed healthcare facilities (UBS), often lacking specific details about the causal agent [22].

To construct and implement the RNS, logistical and information systems must be established to enhance access and support care coordination across different points of care. Well-organized and articulated referral and counter-referral systems can facilitate integration between health services and professionals, improving the sharing of information. PHC is considered strategic for this purpose as it serves as the main entry point to the health system and the coordinator of care within the RNS [19, 20].

As a limitation for carrying out this study, the scarce scientific literature focused on the work of professionals stands out, regarding care for riverside farmers who use pesticides, as well as the development of this care in a network format, which made it difficult to discuss with the data obtained. A vast literature was found about the damage to health and weaknesses of being a riverside farmer who uses pesticides, focusing on the impact on health, the changes for the proper use of personal protective equipment, adaptation and self-care, which greatly helped in this research [14, 17, 23].

Another limitation of the research is that it did not include studies from other countries or experiences related to healthcare management with farmers exposed to pesticides in different countries. Despite our efforts, we were unable to find similar articles in the reviewed databases. For detailed, country-specific information on the use of pesticides in healthcare management, it is recommended to consult the official websites of regulatory bodies, such as the EPA in the United States or relevant national authorities in specific countries [24].

The use of pesticides in healthcare management can vary from country to country based on specific regulations, practices, and healthcare needs. It is important to note that pesticide regulations and practices may change over time, so it is advisable to refer to the most recent sources for up-to-date information on specific countries [25]. While Brazil has specific considerations compared to other regions and countries, this proposed care model can be considered for similar research and analysis of care models used in other countries.

For instance, in the United States, the Environmental Protection Agency (EPA) regulates the use of pesticides, including those used in healthcare settings. Pesticides undergo rigorous testing and evaluation before they are approved for use. Healthcare facilities in the US may utilize pesticides for pest control, disinfection, and prevention of vector-borne diseases [24].

The European Union (EU) has strict regulations regarding pesticide use, including in healthcare settings. The EU establishes maximum limits for pesticide residues in food and provides guidelines for safe pesticide use. Integrated Pest Management (IPM) approaches, which aim to minimize pesticide use and prioritize non-chemical methods, are encouraged in healthcare facilities [26].

The use of pesticides in healthcare management in developing countries can vary significantly. Limited resources and infrastructure in some cases may present challenges in implementing strict regulations or adopting alternative pest management strategies. Nevertheless, efforts are being made globally, according to WHO [25], to promote sustainable pest control practices and reduce reliance on pesticides in healthcare settings.

It is crucial to prioritize the health and safety of patients, healthcare professionals, and the environment when using pesticides in healthcare management. Proper training, risk assessment, and compliance with regulations and best practices are essential for minimizing potential risks associated with pesticide use.

However, the view of health professionals about the implications that involve care for riverside farmers who use pesticides is still incipient in the literature. Another limitation was that the survey was carried out only at the RNS outpatient care points, not including the hospital environment. It is important to investigate the care given to riverside farmers who use pesticides at the tertiary health care level [19].

Studies dealing with the theme would be relevant, aiming at understanding and defining the role of this level in the care of riverside farmers who use pesticides as a Man-Environment process [27], as well as its integration with existing services in the RNS. It is suggested that further research be conducted on the topic of depression caused by pesticides and care management. Furthermore, it is also necessary to think about the use of other methodological approaches that include data of a quantitative nature. Finally, the importance of conducting research focused on the RNS theme in general should be highlighted, thought of as an organizational model for health systems that intend to be integrated.

## Conclusions

The scope of Care Management for riverside farmers who use pesticides from the perspective of the attention of the National Health Networks was affirmed, in this study, as the strategy for the implementation of qualified care for this population group, which demands specific attention, in the different levels of the health system and greater preparation of Family Health Teams, and access to diagnostic tests as well as clinical specialties. The lack of knowledge on the part of its professionals in primary care and the lack of management on the part of municipalities and states for the diagnosis and monitoring of damage caused by pesticides, as well as the little notification of cases, limiting the effectiveness of actions and the discontinuity of care in communities and injuries caused by pesticides. Scientific research to find the specific cause (the type of pesticide) is one of the most important needs to be resolved within the network. This study contributes within science by expanding the theoretical and practical knowledge of the operation of care management, as well as offering new approaches to the study of human behavior and the relationship of the society that governs it.

## Data Availability

All data generated or analyzed during this study are included in this published article. The sets of each interview during the current research are not publicly available, due to the fact that in the clause of the consent of the interview they were agreed as confidential and the answers, because each one is from an area within the municipal health in the researched municipalities, it is possible to identify the interviewees in the responses. But they are available from the corresponding author upon reasonable request. These data supporting the findings of this study are available at the State University of Bahia - UNEB CAMPUS VIII, but there are restrictions on the availability of these data, which were used under license for the current study and therefore are not publicly available. However, the data are made available by the authors upon reasonable request and with permission from the State University of Bahia - UNEB CAMPUS VIII.

## Abbreviations

APS: Primary Health Care
AS: Secondary Care
BA: Bahia’s Municipality, Brazil
CEREST: The establishment of Specialized Reference Centers in Workers’ Health
CHA: Community Health Agents
CM: Care Management
EPA: Environmental Protection Agency
ESF: Family Health Teams in Brazil
GM/MS: Ministry of Health Minister’s Office
CGT: Constructivist Grounded Theory
GT: Grounded Theory
PE: Pernambuco’s Municipality, Brazil
PHC: Primary health Care
PPE: Personal Protective Equipment
RAS: Healthcare network
RENAST: Integral Worker Health Care
RNS: National Health Network in Brazil
SSM: Municipal Health Department
SUS: Unified Health System in Brazil
UBS: The Basic Health Units in Brazil
UNEB: State University of Bahia
UPAs: Urgent Care Units

## References

[1] Gonçalves AB, Cidade EC, da Costa Araujo AC, da Costa TF (2022). Saúde do Trabalhador na Atenção Básica:(des) conhecimento, fragilidades e potencialidades segundo profissionais da Atenção Básica no município de Iguatu/CE. Rev Conjecturas.

[2] Vianna ADS, Câmara VDM, Barbosa MCDM, Santos ADSE, Asmus CIRF, Luiz RR (2022). Exposição ao mercúrio e anemia em crianças e adolescentes de seis comunidades da Amazônia Brasileira. Cien Saude Colet.

[3] Follet C. O veneno está no ar: Um estudo de caso de derivas por agrotóxicos em Nova Santa Rita, Rio Grande do Sul. Cadernos de Agroecologia. 2022; 17(3). Available at http://cadernos.aba-agroecologia.org.br/cadernos/article/view/6795/5049. Accessed 12 Sept 2022.

[4] Amorim LDA, Silva TL, Faria HPD, Machado JMH, Dias EC (2017). Vigilância em saúde do trabalhador na atenção básica: aprendizagens com as equipes de saúde da família de João Pessoa, Paraíba, Brasil. Ciên Saúde Colet.

[5] BRAZIL (2016). Portaria nº 204, de 17 de fevereiro de 2016. Define a Lista Nacional de Notificação Compulsória de doenças, agravos e eventos de saúde pública nos serviços de saúde públicos e privados em todo o território nacional, nos termos do anexo, e dá outras providências. Diário Oficial da União.

[6] BRAZIL. (1989, July 11). Brasília, Lei nº 7.802, de 11 de julho de 1989. Dispõe sobre a pesquisa, a experimentação, a produção, a embalagem e rotulagem, o transporte, o armazenamento, a comercialização, a propaganda comercial, a utilização, a importação, a exportação, o destino final dos resíduos e embalagens, o registro, a classificação, o controle, a inspeção e a fiscalização de agrotóxicos, seus componentes e afins, e dá outras providências. Diário Oficial da União; 1989. Available at https://legislacao.presidencia.gov.br/atos/?tipo=LEI&numero=7802&ano=1989&ato=501MTR61EeFpWT452. Accessed 12 Sept 2022.

[7] Pernambuco. Lei Estadual 12.753, de 21 de janeiro de 2005. Dispõe sobre o comércio, o transporte, o armazenamento, o uso e aplicação, o destino final dos resíduos e embalagens vazias, o controle, a inspeção e a fiscalização de agrotóxicos, seus componentes e afins, bem como o monitoramento de seus resíduos em produtos vegetais, e dá outras providências. Diário Oficial de Pernambuco; 2005. Retrieved September 12, 2022, from https://www.legisweb.com.br/legislacao/?id=333311. Accessed 12 Sept 2022.

[8] Bahia. Lei Estadual 6455 de 25 de janeiro de 1993. Dispõe sobre o controle da produção, da comercialização, do uso, do consumo, do transporte e armazenamento de agrotóxicos, seus componentes e afins no território do Estado da Bahia e dá outras providências. Diário Oficial da Bahia; 1993. Available at https://governo-ba.jusbrasil.com.br/legislacao/86059/lei-6455-93. Accessed 12 Sept 2022.

[9] Silverio AM, Pinheiro PB (2019). A biociência dos agrotóxicos e seu impacto na saúde. Revista Ouricuri.

[10] De Albuquerque Junior EC, Dos Santos Rodrigues HO (2022). Ocorrência do herbicida Diuron no sedimento de rios da sub-bacia do Rio Botafogo, litoral norte de Pernambuco. Rev Biotecnol Ciên.

[11] De Souza JP, De Jesus Porto M, Credidio GC, Teles ALB (2022). Comercialização e utilização de agrotóxicos no município de Crisópolis-BA. Res Soc Dev.

[12] IBGE- BRAZILIAN INSTITUTE OF GEOGRAPHY AND STATISTICS. Portal Cidades. Cidades e Estados. Petrolina e Juazeiro10 de junho de 2022. 2022. Available at http://www.ibge.gov.br. Accessed 12 Sept 2022.

[13] CNES - NATIONAL REGISTRY OF HEALTH ESTABLISHMENTS IN BRAZIL. Estabelecimentos de saúde das cidades Petrolina e Juazeiro do dia 13 de fevereiro 2022. 2022. Available at http://www.cnes.datasus.gov.br. Accessed 13 Sept 2022.

[14] BRAZIL - Secretary of Health Surveillance. Notifiable Diseases Information System (Sinan-Net). Notifications per year, of injuries due to exogenous poisoning caused by agricultural pesticides, in the municipalities of Juazeiro/BA and Petrolina/PE, in the period 2007–2022, with data up to May 2022. 2022. Available at http://www.saude.gov.br/sinan_net. Accessed 13 Sept 2022.

[15] Charmaz KA. A construção da teoria fundamentada: guia prático para análise qualitativa. In: Caregnato SE, coordenador. Métodos de pesquisa. Porto Alegre: Artmed Editora AS; 2009. Available at https://books.google.com.br/books?hl=ptBR&lr=&id=offC0wDYzC4C&oi=fnd&pg=PA6&dq=15.%09Charmaz+KA.+(2009).+A+constru%C3%A7%C3%A3o+da+teoria+fundamentada:+guia+pr%C3%A1tico+para+an%C3%A1lise+qualitativa.+In:+Caregnato+SE,+coordenador.+M%C3%A9todos+de+pesquisa.+Porto+Alegre:+Artmed+Editora+AS.&ots=PNltZ86GK&sig=SxTwdJGIpR63fU63PHqAOccl_U&redir_esc=y#v=onepage&q&f=false. Accessed 16 Sept 2021.

[16] Mendes EV (2010). As redes de atenção à saúde. Ciên Saúde Colet.

[17] Cecílio LCO (2011). Apontamentos teórico-conceituais sobre processos avaliativos considerando as múltiplas dimensões da gestão do cuidado em saúde. Interface-Comunicação: Saúde.

[18] Fenner ALD, Maioli OLG, Machado JMH, Souza MDSD, Machado ADA, Lima ASG, et al. Saúde dos povos e populações do campo, da floresta e das águas: a Fiocruz e sua atuação estratégica na temática de saúde e ambiente relacionada aos povos e populações do campo, da floresta e das águas. FIOCRUZ; 2018. p. 10–109. Available at https://www.arca.fiocruz.br/handle/icict/43275. Accessed 16 Sept 2022.

[19] Ristow LP, Battisti IDE, Stumm EMF, Montagner SED (2020). Fatores relacionados à saúde ocupacional de agricultores expostos a agrotóxicos. Saúde Soc.

[20] BRAZIL. Portaria de Consolidação nº 3 de 14 de junho de 2017. Diretrizes para Organização da Rede de Atenção à Saúde do SUS. Diário Oficial da União; 2017. Available at https://bvsms.saude.gov.br/bvs/saudelegis/gm/2017/MatrizesConsolidacao/Matriz-3-Redes.html. Accessed 14 Sept 2022.

[21] Da Silva Nina LN, Rabelo PPC, de Oliveira BLCA, Caldas ADJM, Rolim ILTP (2022). Atenção primária à saúde e redes de atenção à saúde: Uma reflexão perante a pandemia. Rev Saúde Colet: Barueri.

[22] Fausto MCR, Giovanella L, Lima JG, Cabral LMDS, Seidl H (2022). Sustentabilidade da Atenção Primária à Saúde em territórios rurais remotos na Amazônia fluvial: organização, estratégias e desafios. Cien Saude Colet.

[23] Da Silva Reis MH, Portugal JKA, Campos GL, de Souza Pereira V, Júnior JCFP, Germano SNF (2021). Características da população ribeirinha de um município do interior do Amazonas. Revista Eletrônica Acervo Saúde.

[24] Friedrich K, Silveira GRD, Amazonas JC, Gurgel ADM, Almeida VESD, Sarpa M. Situação regulatória internacional de agrotóxicos com uso autorizado no Brasil: potencial de danos sobre a saúde e impactos ambientais. Cad Saúde Pública. 2021;37. 10.1590/0102-311X00061820. 10.1590/0102-311X00061820 34008735

[25] WHO World Health Organization. FAO/WHO International Code of Conduct on Pesticide Management. Guidance on good pesticide labeling practices (Second revision) – International Code of Conduct on Pesticide Management. Pomegranate. Geneva: World Health Organisation; 2022. Available at https://www.who.int/publications/i/item/9789240053014. Accessed 1 Jan 2023.

[26] McGinley J, Healy MG, Ryan PC, O’Driscoll H, Mellander PE, Morrison L (2023). Impact of historical legacy pesticides on achieving legislative goals in Europe. Sci Total Environ.

[27] De Avila-Pires FD. Princípios de Ecologia Humana: ecologia, sociedade e saúde (Ebook). 2nd ed. Florianópoli; 2020. Available at https://d1wqtxts1xzle7.cloudfront.net/65222960/Ecologia_humana_2020_2ed_AVILA_PIRES-libre.pdf?1608392169=&response-content-disposition=inline%3B+filename%3DEcologia_humana_2020_2ed_AVILA_PIRES.pdf&Expires=1671125776&Signature=Z14fiSFG8HAgDzl2NE3bRmfN43TGFbTrPaDLvqsV5fkMaRabycqlg6uMQM83JOna1AJyoBlDbgl4~KNlTE4XGqc9A7jOIr0KnVnpYeiFY8Ew8vQbdAmR0G8S0O-~a7D8FCL72CQgEoshnIC1KMhDdLdQKkmTgWgQ37jxpMAeHFhfhxdCmHnSQkXwvf1ebG~WLqmJMUm9LgOVJL32WzifFUT5derWzsWRoVL6O2GEAFZUbkapQdiSfmYgQHQ6lq-u6r19jHZuwyZZ962BYW-a9pEf2fRVzu-anVCVyHd2QPhBIKyAaACWvrt0bskzjQABI37ssfKvtYyhoZPQU7xvDA__&Key-Pair-Id=APKAJLOHF5GGSLRBV4ZA. Accessed 16 Sept 2022.

